# The Role of Macrophages in Kidney Fibrosis

**DOI:** 10.3389/fphys.2021.705838

**Published:** 2021-08-06

**Authors:** Xiaoling Wang, Jianwei Chen, Jun Xu, Jun Xie, David C. H. Harris, Guoping Zheng

**Affiliations:** ^1^Shanxi Key Laboratory of Birth Defect and Cell Regeneration, Shanxi Medical University, Taiyuan, China; ^2^Clinical Laboratory, Shanxi Academy of Traditional Chinese Medicine, Taiyuan, China; ^3^Centre for Transplant and Renal Research, Westmead Institute for Medical Research, The University of Sydney, Sydney, NSW, Australia; ^4^Department of General Surgery, First Hospital of Shanxi Medical University, Taiyuan, China

**Keywords:** macrophages, fibrosis, signaling pathways, TGF-β, Wnt

## Abstract

The phenotypic heterogeneity and functional diversity of macrophages confer on them complexed roles in the development and progression of kidney diseases. After kidney injury, bone marrow-derived monocytes are rapidly recruited to the glomerulus and tubulointerstitium. They are activated and differentiated on site into pro-inflammatory M1 macrophages, which initiate Th1-type adaptive immune responses and damage normal tissues. In contrast, anti-inflammatory M2 macrophages induce Th2-type immune responses, secrete large amounts of TGF-β and anti-inflammatory cytokines, transform into αSMA+ myofibroblasts in injured kidney, inhibit immune responses, and promote wound healing and tissue fibrosis. Previous studies on the role of macrophages in kidney fibrosis were mainly focused on inflammation-associated injury and injury repair. Apart from macrophage-secreted profibrotic cytokines, such as TGF-β, evidence for a direct contribution of macrophages to kidney fibrosis is lacking. However, under inflammatory conditions, Wnt ligands are derived mainly from macrophages and Wnt signaling is central in the network of multiple profibrotic pathways. Largely underinvestigated are the direct contribution of macrophages to profibrotic signaling pathways, macrophage phenotypic heterogeneity and functional diversity in relation to kidney fibrosis, and on their cross-talk with other cells in profibrotic signaling networks that cause fibrosis. Here we aim to provide an overview on the roles of macrophage phenotypic and functional diversity in their contribution to pro-fibrotic signaling pathways, and on the therapeutic potential of targeting macrophages for the treatment of kidney fibrosis.

## Introduction

Kidney fibrosis is an inevitable outcome of all progressive chronic kidney diseases (CKD), including hypertensive, diabetic, and vascular nephropathy. Chronic inflammation is a direct cause of kidney injury. Chronic inflammation leads to excessive kidney repair and consequent kidney fibrosis and thereby failure of kidney function. Macrophages have long been known to be master players in inflammatory kidney diseases and to be associated with the development of kidney fibrosis in CKD. However, evidence for a direct contribution of macrophages to kidney fibrosis is lacking. Here we summarize the biological and pathological functions of macrophages polarized during the course of disease progression and their role in the development of kidney fibrosis in CKD, in particular, their contribution to profibrotic signaling networks.

## Macrophage as a Master Player in Kidney Fibrosis

Macrophages are an important part of the mononuclear phagocyte system comprising monocytes, macrophages, and dendritic cells (Viehmann et al., 2018). Mouse F4/80 or human epidermal growth factor module-containing mucin-like receptor 1 (EMR1) are considered to be signature markers of macrophage (Khazen et al., 2005). Macrophages are primarily responsible for pathogen clearance and the repair of injured tissues (Rosenberger and Finlay, 2003; Das et al., 2015). They are multifunctional cells with great phenotypic plasticity serving at the frontier of innate immune defenses. Kidney macrophages include long-lived tissue-resident macrophages and macrophages derived from circulating monocytes of bone marrow origin (Tang et al., 2019). With functional diversity depending on the local microenvironment, macrophages play a critical role in inflammatory kidney disease (Wang and Harris, 2011).

Kidney fibrosis develops in a milieu of inflammatory cell infiltration, mesenchymal cell proliferation and activation, and progressive deposition of extracellular matrix (ECM), leading to scar formation (fibrosis) that destroys the parenchymal structure of kidney and causes progressive loss of kidney function. Observations from human CKD and experimental CKD models have shown that tubulointerstitial fibrosis is an essential feature of chronic kidney failure, and the degree of macrophage infiltration is directly associated with the severity of fibrosis (Yu et al., 2010). Accumulation of kidney macrophages correlates with severity of kidney injury and kidney fibrosis in human and experimental diabetic nephropathy (Chow et al., 2004) and also in other classically non-inflammatory kidney diseases. The infiltration of monocytes expressing chemokine (C-C motif) receptor 2 (CCR2) leads to kidney inflammation and fibrosis in murine chronic obstructive nephropathy (Braga et al., 2018). Kidney macrophage numbers and chemokine (C-C motif) ligand 2 (CCL2) levels correlate significantly with the progression of interstitial fibrosis in human CKD (Eardley et al., 2008). Moreover, selective depletion of macrophages reduces kidney fibrosis (Furuta et al., 1993). These studies support a role for macrophages in genesis and progression of kidney inflammation and fibrosis.

In CKD, macrophages polarize to various phenotypes in response to complex microenvironmental stimuli in diseased kidneys. Macrophages of different phenotypes secrete a variety of growth factors, cytokines, proteins, and enzymes which contribute to or mitigate fibrosis (Eddy and Neilson, 2006). Macrophages produce profibrotic mediators including TGF-β, Wingless and Int-1 (Wnt), platelet-derived growth factor (PDGF), tumor necrosis factor α (TNF-α), hepatocyte growth factor (HGF), connective tissue growth factor (CTGF), angiotensin converting enzyme (ACE), angiotensin I (Ang I) and II (Ang II), plasminogen activators, plasminogen activator inhibitor-1 (PAI-1), tissue inhibitor of metalloproteinases (TIMP), collagen, fibronectin, thrombospondin, coagulation factors, reactive oxygen species, and endothelin. They can also produce mediators that protect against kidney fibrosis including collagenases, matrix metalloproteinase 12 (MMP-12), nitric oxide, and bone morphogenic protein-7 (BMP-7) (Eddy, 2011). Macrophages of various phenotypes are therefore responsible for several key processes in progressive fibro-inflammatory kidney disease, including initiation of inflammatory damage, resolution of inflammation, phagocytotic clearance of debris after inflammation, tissue repair, remodeling of fibrotic tissue, and excessive repair leading to irreversible kidney fibrosis. Thus, macrophages play very complex roles in kidney fibrosis (Ricardo et al., 2008; Shen et al., 2014; Table 1).

**TABLE 1:** Macrophage phenotypes, stimuli, Secreted products, and functions.

Macrophage phenotypes	Stimuli	Secreted products	Phenotypic function
M1	LPS (Kalish et al., 2015), TNF-α (Venturin et al., 2016), IFN-γ (Lee et al., 2011), S100A9 (Tang et al., 2019), IL-1α (Tang et al., 2019)	IL-1 (Tang et al., 2019), IL-1β (Wong et al., 2018), IL-6 (Tang et al., 2019), IL-8 (Kadowaki et al., 2009), IL-12 (Tang et al., 2019), IL-17A (Wong et al., 2018), IL-23 (Tang et al., 2019), TNF-α (Inoue, 2017), iNOS (Inoue, 2017), MMP-12 (Tang et al., 2019), CCL-2 (Wong et al., 2018), CCL-3 (Meng et al., 2015), CCL-5 (Meng et al., 2015), CXCL1 (Meng et al., 2015), CXCL2 (Meng et al., 2015), CXCL10 (Meng et al., 2015), ICAM-1 (Wong et al., 2018), Wnt5a (Blumenthal et al., 2006) RAS (Ulrich et al., 2011)	Pro-inflammatory and TH1-like immune response (Tang et al., 2019)
M2a	IL-4 (Zhang et al., 2017; Tang et al., 2019), IL-13 (Zhang et al., 2017; Tang et al., 2019)	Mannose and scavenger receptor (Anders and Ryu, 2011), decoy IL-1R11 (Anders and Ryu, 2011), FIZZ1 (Anders and Ryu, 2011), YM-1 (Anders and Ryu, 2011), IL-10 (Lu et al., 2013), TGF-β (Lu et al., 2013), Wnt1, Wnt3a (Cosin-Roger et al., 2019), Wnt7b (Lin et al., 2010) CCL13 (Meng et al., 2015), CCL14 (Meng et al., 2015), CCL17 (Meng et al., 2015), CCL18 (Meng et al., 2015), CCL22 (Meng et al., 2015), CCL23, CCL24 (Meng et al., 2015), CCL26 (Meng et al., 2015), MMP-9 (Meng et al., 2015), MMP-12 (Meng et al., 2015), IGF-1 (Meng et al., 2015), arginase 1 (Tseng et al., 2020), Fibronectin (Tseng et al., 2020)	Anti-inflammatory TH2-like immune response (Tang et al., 2019), wound healing and tissue fibrosis (Tang et al., 2019), inhibition of T-cell proliferation (Lu et al., 2013)
M2b	Immune complexes (IgG4) (Bianchini et al., 2019; Tang et al., 2019), TLR/IL-1R ligand (Tang et al., 2019), IL-1R (Lisi et al., 2014), IgG Fc receptor ligands (Lisi et al., 2014), CD40 (Lisi et al., 2014), IL-6 (Philipp et al., 2018)	IL-1 (Anders and Ryu, 2011), IL-6 (Anders and Ryu, 2011), TNF-α (Anders and Ryu, 2011), MHCIIhi (Anders and Ryu, 2011), IL-10hi (Anders and Ryu, 2011), IL-12lo (Anders and Ryu, 2011), IL-1β (Wang et al., 2019), MCP-1 (Chen et al., 2013), iNOS (Chen et al., 2013)	Immunoregulation (Tang et al., 2019), Th2 activation (Meng et al., 2015)
M2c	IL-10 (Tang et al., 2019), TGF-β (Tang et al., 2019), glucocorticoids (Tang et al., 2019)	IL-10 (Anders and Ryu, 2011), TGF-β (Anders and Ryu, 2011), mannose receptor (Anders and Ryu, 2011), B7-H4 (Lu et al., 2013), arginase 1 (Meng et al., 2015)	Immunosuppression (Tang et al., 2019), matrix remodeling and tissue repair (Tang et al., 2019), inhibition of T-cell proliferation (Lu et al., 2013), induction of Tregs (Lu et al., 2013)

*PAMPs, pathogen-associated molecular patterns; DAMPs, danger- associated molecular patterns; FGF2, basic fibroblast growth factor; LPS, lipopolysaccharide; TLR, toll-like receptors; TNF, tumor necrosis factor; iNOS, inducible nitric oxide synthase; MINCLE, macrophage-inducible C-type lectin; Arg1, arginase-1; MCHII, major histocompatibility complex (MHC) class II; MR, mannose receptor; IGF-1, insulin like growth factor; IRF, interferon-related factor; IL-1R, IL-1 receptor; TGF-β, transforming growth factor-β; Wnt, Wingless and Int-1; RAS, renin angiotensin system.*

## Macrophage Contributions to Kidney Fibrosis Via Inflammation

Inflammation, starting from recruitment and activation of macrophages, is considered to be a key factor behind fibrotic diseases (Cao et al., 2015). Macrophages are rapidly recruited to the glomerulus or tubulointerstitium to initiate innate immune responses and play important defensive as well as destructive roles in kidney injury. Ongoing kidney damage can cause continuing macrophage infiltration in a vicious cycle that leads to destruction of the normal kidney tissue structure and irreversible tissue fibrosis. Although it is widely believed that glomerular and interstitial macrophages are closely associated with development of kidney fibrosis, they also play beneficial roles in stromal remodeling during tissue repair (Ricardo et al., 2008; Alikhan and Ricardo, 2013). It is important to understand the complex roles of macrophages in kidney inflammation and fibrosis.

### Inflammatory Role of M1 Macrophages

**FIGURE 1:**
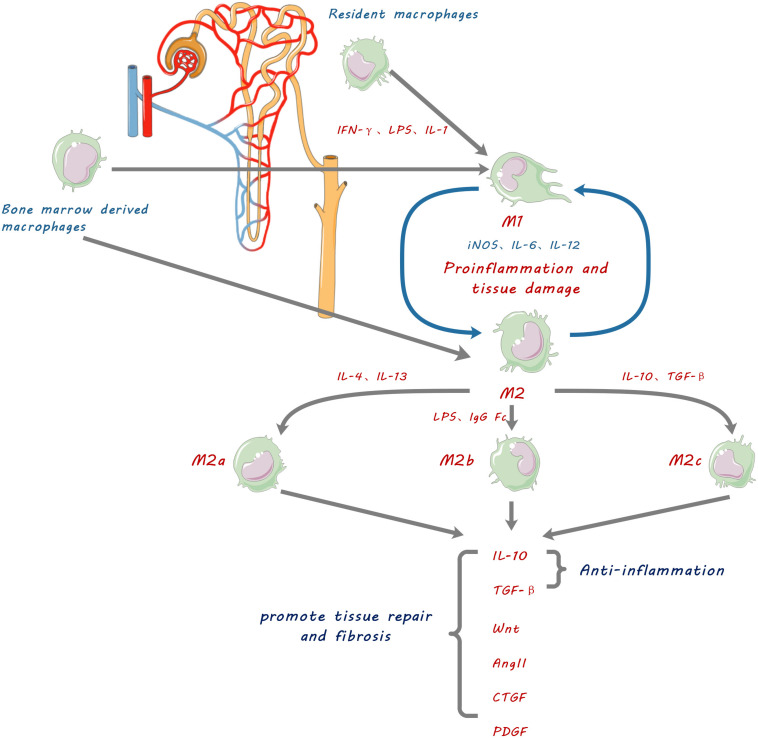
Schematic diagram showing macrophage contribution to kidney fibrosis. Kidney resident macrophages and infiltrating bone marrow-derived macrophages are stimulated by inflammatory factors such as IFN-γ, lipopolysaccharide (LPS), and IL-1, to be polarized into pro-inflammatory M1 macrophages which promote inflammation and tissue damage by releasing IL-6, 12 and inducible nitric oxide synthase (iNOS). They can also be polarized into three functionally different phenotypes of anti-inflammatory and reparative M2 macrophages by anti-inflammatory cytokines IL-4, IL-10, IL-13, and TGF-β. M2 macrophages resolve inflammation, promote tissue repair and cause fibrosis by secretion of anti-inflammatory cytokines and tissue repair mediators including IL-10, transforming growth factor.(TGF-β), Wingless and Int-1 (Wnts), Angiotensin II (Ang II), connective tissue growth factor (CTGF), and platelet-derived growth factor (PDGF).

The ability of macrophages to play complex roles in kidney diseases is explained by their phenotypic heterogeneity and functional diversity (Anders and Ryu, 2011). Macrophages are activated and differentiated under specific microenvironmental conditions into two broad phenotypes, namely classically activated macrophages (CAM or M1) and alternatively activated macrophages (AAM or M2) (Figure 1). However, the concept of M1 and M2 macrophage phenotypes was mostly derived from *in vitro* observations of cultured macrophages. Such distinct M1 and M2 macrophage phenotypes are not consistent with *in vivo* observations, where M1 and M2 markers can co-exist on same macrophage (Wang et al., 2014). We use the terms of M1 and M2 macrophage phenotypes in this review for the convenience in citing respective studies and for description of functionally different macrophages. The existence of such heterogeneous phenotypes is explained by the cellular plasticity of circulating monocytes and macrophages in response to different stimuli. There is compelling evidence that the major factor determining kidney injury versus tissue restoration is the activation state of macrophages within local tissues rather than the degree of macrophage infiltration (Ricardo et al., 2008).

Circulating monocytes are recruited by cytokines and chemoattractants within the pathogenic microenvironment of diseased kidneys. They adhere to activated endothelial surfaces, infiltrate into interstitial and/or glomerular compartments, and differentiate into pro-inflammatory M1 macrophages (Tang et al., 2019). M1 macrophages can be polarized by pathogen-related molecular patterns (PAMPs) such as lipopolysaccharides (LPS), alarmins such as S100A9 and IL-1α, and pro-inflammatory cytokines such as tumor necrosis factor α (TNF-α) (Kalish et al., 2015; Venturin et al., 2016). Polarized M1 macrophages highly express major histocompatibility complex (MHC) class II and co-stimulating molecule CD86 and initiate Th1 type adaptive immune responses, resulting in cytotoxicity and more effective killing of bacteria, intracellular pathogens and tumor cells (Lv et al., 2017; Tang et al., 2019). Concurrently, M1 macrophages secrete a series of pro-inflammatory factors (including IL-1, IL-6, IL-12, TNF-α), chemokines (such as IL-8), activated oxygen species, and nitric oxide (NO) which promote inflammation and damage of normal tissues (Inoue, 2017; Tang et al., 2019).

In the early stage of kidney ischemia-reperfusion injury (IRI) in rats, macrophages are M1 in phenotype and highly express iNOS (Huen and Cantley, 2015). Depletion of macrophages at this stage by liposome clodronate significantly attenuated kidney injury, accompanied with decreased expression of inflammatory and profibrotic cytokines (Ko et al., 2008). Similarly, miR-30c-5p agomir which directly inhibits Interferon regulatory factor 1 (IRF1) reduced kidney ischemic injury by reducing M1 macrophages and increasing of M2 macrophages, and by reducing inflammatory cytokine TNF-α and increasing anti-inflammatory cytokines IL-4 and IL-10 (Zhang et al., 2019; Guo et al., 2020). In contrast, transfusion of IFN- induced M1 macrophages following acute kidney IRI increased tubulointerstitial fibrosis and functional impairment (Lee et al., 2011).

Apart from inflammatory cytokines, activated macrophages also secrete matrix metalloproteinases (MMPs), including abundant MMP- 1, −3, −7, −9, −10, −12, −14, and −25 with less abundant MMP-2, 3, 8, 10, 11, 12 (Huang et al., 2012). Those MMPs contribute not only to degradation of extracellular matrix, but also to inflammatory injury in kidney (Kunugi et al., 2011). Macrophage-derived MMP-9 has been shown to contribute to kidney fibrosis through induction of profibrotic changes in tubular epithelial cells (Tan et al., 2010) and recruitment of macrophages via proteolytic activation of osteopontin (Tan et al., 2013). More importantly, MMP-mediated proteolytic releasing and activation of TGF-β bound to extracellular matrix (Karsdal et al., 2002) may directly contribute to kidney fibrosis and indirectly through induction of profibrotic M2 macrophages.

In addition to promotion of inflammation and tissue damage, pro-inflammatory M1 macrophages were found also to be capable of switching to anti-inflammatory and reparative M2 macrophages (Arnold et al., 2007). Thus, the classification of M1 and M2 macrophage phenotypes may well represent but oversimplify the plastic functional status of macrophages at different stages of disease progression.

### Anti-inflammatory and Pro-fibrotic Roles of M2 Macrophages

Alternatively activated macrophages, M2 macrophages, can be defined from *in vitro* experiments into three functional subtypes according to their activation stimuli and functions: M2a, M2b, and M2c (Tang et al., 2019; Figure 1). M2a macrophages are typically induced by IL-4 and IL-13 (Zhang et al., 2017); M2b macrophages are induced by immune complexes, LPS, IgG Fc receptor ligands, and CD40 (Lisi et al., 2014); M2c macrophages are induced by IL-10 and TGF-β or glucocorticoids (Kim et al., 2015). Those phenotypic definitions of anti-inflammatory M2 macrophages are used for the convenience in description of their respective functions.

The subtypes of M2 macrophages are thought to suppress immune responses and promote tissue repair, but with different and sometimes controversial functions (Mantovani et al., 2004). M2a macrophages, highly express the marker arginase 1 (Arg-1), produce a large amounts of anti-inflammatory IL-10 and IL-1 receptor antagonist (IL-1ra), and inhibit secretion of pro-inflammatory cytokines (IL-12, IL-1, TNF-α) and production of NO, thereby exerting anti-inflammatory and immunosuppressive functions. M2b macrophages specifically up-regulate IL-10 and down-regulate IL-12, and induce T cells to secrete IL-4, which in turn promotes B cells to produce antibodies, and induce anti-inflammatory Th2 immune responses. M2c macrophages secrete large amounts of IL-10 and TGF-β, suppress inflammatory immune responses, and promote wound healing and tissue fibrosis (Tang et al., 2017, 2019). Supporting evidence includes that reduced infiltration of macrophages (mainly M2) in murine models of kidney disease can prevent progressive interstitial collagen deposition and inhibit kidney fibrosis (Kim et al., 2015). Furthermore, the adoptive transfer of M2c macrophages rather than M1 macrophages reversed the beneficial effects of macrophage depletion in kidney fibrosis (Tang et al., 2019). In the unilateral ureteral obstruction (UUO) model, depletion of macrophages from day 4 significantly reduced kidney fibrosis, while the adoptive transfer of M2 macrophages promoted the accumulation of αSMA+ cells and kidney fibrosis (Shen et al., 2014). In a rat model of anti-glomerular basement membrane disease, inhibition of M2 macrophage infiltration by inhibitor of the macrophage-specific c-fms receptor at days 14–35 resulted in a significant reduction in both glomerular sclerosis and interstitial fibrosis (Han et al., 2013). Consistent with findings from experimental animal models, the number of M2 macrophages expressing CD206 and/or CD163 is associated with kidney interstitial fibrosis and tubular atrophy in human kidney diseases such as diabetic nephropathy, IgA nephropathy, and in kidney transplants (Wu et al., 2020). Together these findings indicate that M2 macrophage polarization and infiltration can promote kidney fibrosis and progression of kidney disease. However, in acute or non-persistent kidney injuries such as acute tubular necrosis (ATN), M2 macrophages were mainly anti-inflammatory and promoted epithelial healing and rapid regeneration of intact tubules (Anders and Ryu, 2011).

M2 macrophages were thought to promote kidney fibrosis via secretion of TGF-β1 which is well-known to cause fibrosis; larger quantities of TGF-β1 were detected in M2 macrophages than in myofibroblasts in the UUO model (Shen et al., 2014). However, macrophage-specific deletion of TGF-β1 failed to prevent renal fibrosis after severe ischemia-reperfusion or obstructive injury (Huen et al., 2013). In contrast, selective deletion of TGF-β receptor II (TβRII) in monocytes/macrophages promoted kidney fibrosis by enhancing renal macrophage infiltration (Chung et al., 2018). These controversial findings suggested that it would be too simplistic to conclude or disprove profibrotic roles of macrophage TGF-β1 by selective depletion of either TGF-β1 or its receptor (TβRII) alone, given that TGF-β1 is also the most potent anti-inflammatory factor secreted by M2 macrophages (Ricardo et al., 2008), and inflammation is unarguably the initial cause of kidney fibrosis (Tang et al., 2019). We found that by alteration of TGF-β1 signaling in bone marrow-derived macrophages via shifting β-catenin binding from TCF to Foxo1 using β-catenin/TCF inhibitor ICG-001, the anti-inflammatory function of TGF-β1 was enhanced by increased production of anti-inflammatory IL-10 and reduced production of IL-6 and TNF-α in the bone marrow-derived macrophages. Concurrently the pro-fibrotic effect of TGF-β1 was abolished by significant reduction of GFP (+) F4/80 (+) α-SMA (+) bone marrow-derived macrophages undergoing macrophage-myofibroblast transformation (MMT) (Wang et al., 2017) and thereby kidney fibrosis was reduced in the murine model of unilateral ureteral obstruction (UUO) (Yang et al., 2019).

In addition to TGF-β1, M2 macrophage polarization is also tightly regulated by the Wnt pathway. Wnt5a can enhance TGF-β-induced macrophage M2 polarization and the expression of Yes-associated protein (Yap)/transcriptional coactivator with PDZ-binding motif (Taz) to promote kidney fibrosis (Feng et al., 2018a). The Wnt ligand Wnt3a induces the polarization of M2 macrophages by enhancing IL-4 or TGF-β1 (Feng et al., 2018b). Conditional deletion of Wnt3a in bone marrow cells lessens the accumulation of macrophages and the polarization of M2, and reduces kidney fibrosis in the murine UUO model (Feng et al., 2018b).

### Bone Marrow Macrophage Contribution to Kidney Fibrosis

Bone marrow-derived monocytes are recruited to the kidney after injury. They constitute a large proportion of interstitial infiltrating macrophages (Tang et al., 2019) and play a major role in progression of kidney fibrosis as they polarize to macrophages of various phenotypes (Conway et al., 2020). Bone marrow-derived macrophages can differentiate into α-SMA+ myofibroblasts in injured kidney, via MMT (Ikezumi et al., 2015; Wang et al., 2017). Flow cytometric analysis found that most CD45+ leukocytes isolated from obstructed kidneys expressed both collagen I and α-SMA (Chen et al., 2011). The CD45+ cells in these fibrotic kidneys are infiltrating monocytes derived from bone marrow. They have undergone MMT and transdifferentiated into collagen-producing myofibroblasts within the microenvironment of the damaged kidney, driven by TGF-β1 (Nikolic-Paterson et al., 2014) secreted by M2 macrophages (Shen et al., 2014). *In vitro* TGF-β1 drove transdifferentiation of cultured macrophages into collagen-secreting α-SMA+ myofibroblasts (Pilling and Gomer, 2012). Cells expressing macrophage marker CD68 and myofibroblast marker α-SMA+ have been identified in the kidney of patients with active fibrosis (Meng et al., 2016b). Nikolic-Paterson et al. (2014) found evidence of MMT in human kidney disease with active fibrosis using confocal microscopy, and showed that the severity of kidney fibrosis correlated with the number of MMT cells co-expressing α-SMA and CD68. In addition to TGF-β1, chemokine receptor CXCR6 contributes to recruitment of bone marrow-derived fibroblast precursors (Xia et al., 2014), while IL-4 and IL-13 activated Jak3/STAT6 signaling stimulates bone marrow–derived fibroblast MMT in the UUO model of kidney fibrosis (Yan et al., 2015; Liang et al., 2017).

The contribution of bone marrow-derived macrophages to kidney fibrosis is also supported by the observation that down-regulation of CCR2 expression reduced recruitment and activation of myeloid derived macrophages and alleviated kidney fibrosis in UUO model (Jiang et al., 2019). Production of chemokine CXCL16 by kidney tubular epithelial cells is necessary for recruitment of myeloid derived CD45+ col I+ α-SMA+ cells and development of kidney fibrosis in UUO model (Chen et al., 2011; Nikolic-Paterson et al., 2014).

## Macrophage Contribution to Profibrotic Signaling Pathways

Kidney fibrosis is the direct result of activation of fibroblasts and accumulation of myofibroblasts, driven by multiple profibrotic signaling pathways (Kuppe et al., 2021). Profibrotic changes in other cells, including mesenchymal transition of tubular epithelial cells (EMT) (Zheng et al., 2009; Tan et al., 2010; Qiao et al., 2018; Yang et al., 2020; Rao et al., 2021) and endothelial cells (EndoMT) (Zeisberg et al., 2008; Li et al., 2010; LeBleu et al., 2013; Zhao et al., 2016), also contribute to the activation of fibroblasts and kidney fibrosis, but may not directly transform into myofibroblasts (Kuppe et al., 2021).

## Wnt/β-Catenin Signaling Pathway

The Wnt/β-catenin signaling pathway is activated in various kidney diseases, contributing to the development and progression of kidney fibrosis (Zhou et al., 2020). Wnt/β-catenin signaling is an evolutionarily conserved pathway involved in embryonic development, tissue homeostasis, and organ injury repair (Ng et al., 2019; Perugorria et al., 2019). Wnt ligands are a large family of secreted glycoproteins and fundamentally indispensable for transduction of the Wnt signaling pathway (Nie et al., 2020).

Wnt/β-catenin signaling in kidney disease is versatile; transient activation of Wnt/β-catenin signaling induces repair and regeneration during acute kidney injury, but sustained (uncontrolled) Wnt/β-catenin activation promotes kidney fibrosis (Schunk et al., 2021). Lin et al. (2010) found that Wnt7b secreted by macrophages facilitates kidney regeneration through directing epithelial cell-cycle progression and basement membrane repair; kidney injury repair was substantially retarded after macrophage specific deletion of Wnt7b.

**FIGURE 2:**
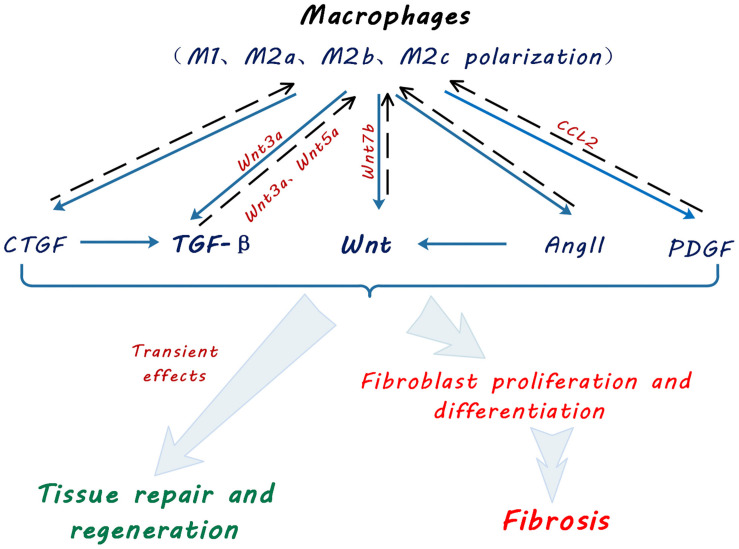
Schematic diagram showing macrophage contribution to profibrotic signaling pathways. Macrophages promote tissue repair, regeneration and fibroblast activation and myofibroblast proliferation via multiple signaling pathways by secretion of transforming growth factor beta (TGF-β), Wingless and Int-1 (Wnts), angiotensin II (Ang II), connective tissue growth factor (CTGF) and platelet-derived growth factor (PDGF). Solid arrows indicate secretion of cytokines and growth factors by macrophages. Broken line arrows indicate autocrine effects of macrophage-secreted cytokines and growth factors on macrophage functional polarization. Light blue arrows indicate effects of macrophage secreted cytokines and growth factors on kidney tissue repair and fibrosis.

In kidney, Wnt5a promotes fibrosis by stimulating Yap/Taz-mediated macrophage polarization in both UUO and IRI models (Feng et al., 2018a). Wnt3a can also promote M2 macrophage polarization induced by IL-4 or TGF-β1, following Wnt/β-catenin signaling activation, and in turn accelerate macrophage proliferation and accumulation, giving rise to kidney fibrosis (Cosin-Roger et al., 2019; Figure 2).

Studies of fibrosis in other organs also demonstrated macrophage contribution to the Wnt/β-catenin pathway. After myocardial infarction in mice, macrophages within the area of infarction exhibited an increase in expression of non-canonical Wnt ligands Wnt5a and Wnt11 (Palevski et al., 2017). The activated Wnt/β-catenin signal promoted cardiac fibrosis by inducing the transition of endothelial cells and epicardial cells to a mesenchymal state, fibroblast differentiation into myofibroblasts and collagen production (Palevski et al., 2017). In a murine model of intestinal fibrosis, CD16+ macrophages expressed high levels of Wnt6, inducing intestinal fibrosis (Salvador et al., 2018). M2 macrophage release of Wnt7a promoted myofibroblast differentiation of lung resident mesenchymal stem cells, leading to lung fibrosis (Hou et al., 2018).

## TGF-β Signaling Pathway

TGF-β is a well-known inducer of kidney fibrosis. While secretion of anti-inflammatory TGF-β by M2 macrophages contributes to resolution of inflammation, it also mediates kidney injury repair and causes kidney fibrosis when in excess. The mechanism by which macrophages promote kidney fibrosis through the TGF-β signaling has been extensively investigated. M1 macrophages can be reprogrammed into alternately activated M2 macrophages by anti-inflammatory cytokine stimulation (IL-10 or colony-stimulating factor 1) or upon their phagocytotic ingestion of apoptotic cells. M2 macrophages promote and coordinate the regeneration of kidney tubular cells and maintain the integrity of the kidney tubules after injury (Rogers et al., 2014). During tissue repair, M2b and M2c macrophages are mainly responsible for immunosuppression, matrix remodeling and wound healing once tissue damage has been resolved (Tang et al., 2019). In contrast, uncontrolled kidney inflammation triggers M2a macrophage polarization in the injured kidney through IL-4 and IL-13, promoting increased TGF-β1 production and kidney fibrosis (Pan et al., 2015). M2 macrophages exert anti-inflammatory effects and promote kidney fibrosis through tissue repair by producing a large amount of TGF-β1 in the UUO model (Eddy, 2005).

## Renin-Angiotensin System (RAS), PDGF and CTGF Signaling Pathways

In addition to Wnt and TGF-β, macrophages are also identified as a source of components of the renin-angiotensin system (RAS), including renin, angiotensin converting enzyme (ACE), Ang I and Ang II, AT1 and AT2 receptors (Okamura et al., 1999). The RAS is known to cause kidney fibrosis through Wnt/β-catenin signaling (Miao et al., 2019; Figure 2). Other pro-fibrotic mediators such as PDGF and CTGF were also found to be produced by macrophages (Cicha et al., 2005; Eitner et al., 2008).

## Integrin/ILK and Notch Signaling Pathways

Apart from direct secretion of pro-fibrotic mediators, macrophages produce matrix metalloproteinases (MMP), which not only contribute to tissue remodeling after injury, but also activate other pro-fibrotic signaling pathways such as Integrin/ILK (Zheng et al., 2009, 2016; Tan et al., 2010) and Notch (Zhao et al., 2016).

## Activation and Proliferation of Myofibroblasts by Crosstalk Between Profibrotic Signaling Pathways

**FIGURE 3:**
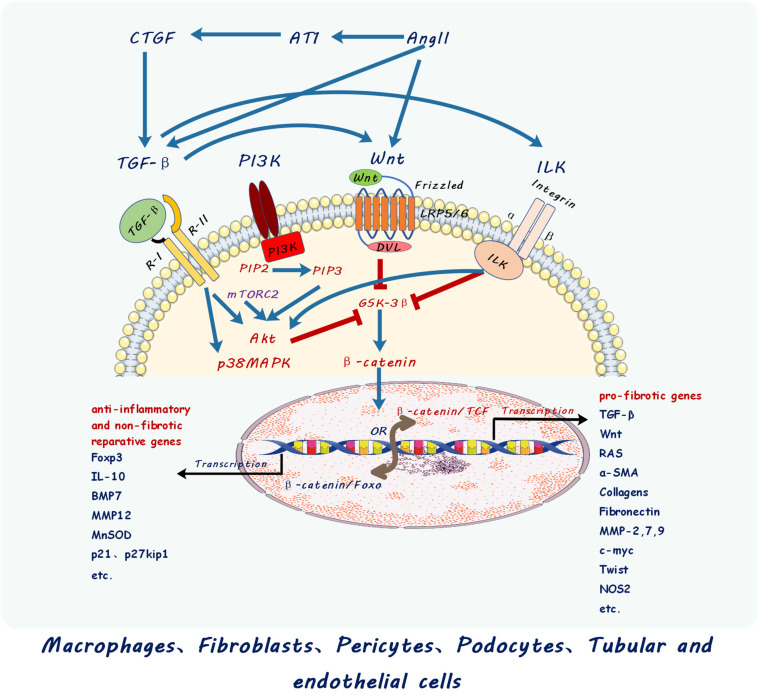
Schematic diagram showing cross-talk between different signaling pathways including those of transforming growth factor beta (TGF-β), Wingless and Int-1 (Wnts), Renin Angiotensin System (RAS), Integrin linked kinases (ILK), connective tissue growth factor (CTGF), and PI3K–mTOR. Multiple signaling pathways cross-talk and converge at β-catenin nuclear translocation and binding with different transcription factors to activate different target genes in macrophage and kidney cells described. PI3K, Phosphatidylinositol 3 kinase; Akt, Ak strain protein kinase B; mTOR, mammalian target of rapamycin; PIP2, phosphatidylinositol (4,5)-trisphosphate; PIP3, phosphatidylinositol (3,4,5)-trisphosphate; mTORC2, mTOR complex 2 including mTOR, Rictor, GβL, Sin1, PRR5/Protor-1, and DEPTOR; Ang II, Angiotensin II; AT1, Angiotensin receptor 1.

Activation and proliferation of myofibroblasts is a central and complex event in development of kidney fibrosis. It involves multiple signaling pathways activated by profibrotic mediators from the fibro-inflammatory microenvironment of the injured kidney. Macrophages are unarguably a major source of those mediators (Table 1). A profibrotic signaling network including TGF-β/Smad, Wnt/β-catenin, the renin- angiotensin system (RAS) and Integrin/ILK pathways cross-talk and synchronize to promote kidney fibrosis (Figure 3).

Wnt/β-catenin signaling is a key player in kidney fibrosis contributing to activation of fibroblasts into myofibroblasts and consequent excessive extracellular matrix production. Upon binding of Wnt ligands to its receptor Frizzled (Fz) and transmembrane receptor LRP5/6, dishevelled (dvl) protein in the cytoplasm is phosphorylated and activated to bind to Axin to antagonize GSK3β, which prevents β-catenin signaling by degradation of cytosolic β-catenin via phosphorylation and ubiquitination machinery (Tan et al., 2014; Wang Y. et al., 2018). Inhibition of GSK3β by Wnt ligands results in β-catenin nucleus translocation followed by transcriptional activation of Wnt target genes when β-catenin complexes TCF/LEF, the transcription binding partners of β-catenin (Zhou et al., 2012; Guo et al., 2019). This canonical Wnt/β-catenin signaling pathway activates a transcriptome of profibrotic inducers such as Snail/Slug, and fibrotic genes such as α smooth muscle actin (α-SMA), collagen, fibronectin and other extracellular matrix genes involved in fibroblast activation and extracellular matrix production. Importantly, Wnt/β-catenin signaling is not acting alone during the development of kidney fibrosis.

TGF-β released by M2 macrophage is also one of the most important contributors to kidney fibrosis. TGF-β signals through both Smad-dependent and Smad-independent pathways. TGF-β binds to TGF-β receptor II which sequentially complexes with TGF-β receptor I. TGF-β receptor II binding to receptor I then leads to receptor I phosphorylation of Smad2/3 which translocate into the nucleus with co-Smad4 to activate profibrotic gene transcription in kidney myfibroblasts (Meng et al., 2016a). In addition to Smad-dependent signaling in activating profibrotic genes in myofibroblasts, TGF-β also promotes β-catenin nuclear translocation through phosphorylation of β-catenin Tyr-654 and dephosphorylation of β-catenin Ser-37 and Thr-41. Furthermore, Smad-independent activation of Akt and p38 MAP kinase (Wang et al., 2011; Zhou et al., 2012; Tan et al., 2014) also subsequently inhibit GSK3β, thereby promoting β-catenin nuclear translocation and activation of Wnt/β-catenin signaling.

The renin-angiotensin system (RAS) is also known to cause hypertension and fibrosis in CKD (Floege, 2015). Macrophage secretion of RAS components [renin, angiotensin converting enzyme (ACE), Ang I and Ang II] promote synthesis and release of profibrotic factors TGF-β, CTGF, PDGF, ET1 (Wang M. et al., 2018; Zhou et al., 2020) and is a direct target of the Wnt/β-catenin pathway, causing kidney injury, and fibrosis. Reciprocally, blockade of Wnt/β-catenin by inhibition of β-catenin/TCF signaling also blocks RAS and consequent hypertension and kidney fibrosis in CKD (Floege, 2015).

Integrin/ILK are known to contribute to both glomerular and interstitial fibrosis in diseased kidneys (Liu, 2010; Zheng et al., 2016). The underlying mechanism for ILK in causing fibrotic signaling involves its direct or indirect (via activation of Akt) inhibition of GSK3β in facilitating β-catenin nuclear translocation and activation of Wnt/β-catenin signaling (Liu, 2010). We found that proximal tubular cell upregulation of ILK via the compensatory increase of α3 integrin worsened kidney fibrosis in the UUO model in proximal tubular specific E-cadherin knockout mice (Zheng et al., 2016). Importantly, ILK is downstream of TGF-β mediation of both glomerular and tubulointerstitial fibrosis in kidneys (Li et al., 2009; Kang et al., 2010). Our study demonstrated that autophagy links TGF-β/Smad signaling with β-catenin through the pY654-beta-catenin/p-Smad2/ILK pathway (Pang et al., 2016).

mTOR activation has been identified in macrophages and myofibroblasts in kidney fibrosis (Chen et al., 2012). mTORC1 activation in podocytes led to the development of glomerular crescents contributing to fibrosis of glomeruli in both experimental and human glomerulonephritis (Mao et al., 2014). mTORC2 is activated by TGF-β to transduce profibrotic signaling through mTOR activation of PI3K-Akt (Li et al., 2015) which subsequently inactivates GSK3β to facilitate β-catenin nuclear translocation and thereby activate β-catenin/TCF in the Wnt/β-catenin pathway. Macrophage polarization has been shown to be controlled by the PI3K-Akt-mTOR pathway; increased mTORC1 activity promoted M1 macrophage polarization and reduced M2 macrophage polarization (Weichhart et al., 2015). mTOR activation was observed in myofibroblasts and macrophages and inhibition of mTOR pathway by rapamycin ameliorated kidney fibrosis (Chen et al., 2012). Both TGF-β and ILK activate PI3K-Akt and thus cross-talk with mTOR, whereas mTORC2 activation of PI3K-Akt also links with the Wnt/β-catenin pathway via PI3K-Akt inhibition of GSK3β (Ching and Hansel, 2010).

Together multiple signaling pathways (TGF-β, Wnt, ILK, RAS, mTOR, etc.) interact via activation of β-catenin in the initiation and progression of kidney fibrosis. The functional status of β-catenin determines the activity of these signaling pathways and the progression or regression of kidney fibrosis. Studies from us and others demonstrated the key role for β-catenin/TCF in mediating profibrotic signaling of multiple pathways (Liu, 2010; Qiao et al., 2018). Importantly, we found that shifting β-catenin binding from TCF toward Foxo in both macrophages and kidney tubular cells by inhibition of β-catenin/TCF redirected TGF-β signaling from pro-fibrotic to anti-inflammatory, protected against kidney fibrosis and promoted epithelial repair in UUO and IRI models (Qiao et al., 2018; Rao et al., 2019, 2021; Yang et al., 2019).

## Targeting Macrophages as a Treatment for Kidney Fibrosis

Anti-inflammatory and reparative properties of macrophages (Lin et al., 2010; Urbina and Singla, 2014; Ratnayake et al., 2021) argue for their therapeutic application. We have shown that *ex vivo* programmed M2 macrophages protect against inflammation and kidney injury in experimental models of inflammatory renal disease (Wang et al., 2007; Cao et al., 2010, 2011). Jung et al. (2012) found that infusion of IL-10 overexpressing macrophages protected ischemia injury in an IRI model. Adoptive transfer of genetically modified macrophages expressing heme-oxygenase-1 (HO-1) protected kidney function in mice with IRI (Ferenbach et al., 2010). Netrin-1-induced M2 macrophages suppressed inflammation and protected against kidney injury in IRI mice (Ranganathan et al., 2013).

However, the phenotypic instability of those M2 macrophages remains as a challenge (Cao et al., 2014). To overcome the hurdle of phenotypic instability, adenovirus vector NGAL (Neutrophil gelatinase-associated lipocalin-2) was used to stabilize phenotype of injected M2 macrophages which reduced inflammation and fibrosis in UUO model. While protection by anti-inflammatory M2 macrophages has been reported increasingly, the profibrotic effects of M2 macrophages remain largely unaddressed as another hurdle for their therapeutic application; M2 macrophages secrete large amounts of TGF-β which not only suppresses inflammation but also promotes kidney fibrosis (Kim et al., 2015).

Depletion of inflammatory M1 macrophages does not protect against kidney fibrosis, while depletion of anti-inflammatory and reparative M2 macrophages can reduce kidney fibrosis (Shen et al., 2014). Thus, although inflammation is an important driver of fibrosis, other non-inflammatory profibrotic pathways are activated by anti-inflammatory and tissue reparative cytokines from M2 macrophages such as TGF-β, Wnt, Ang II, CTGF, and PDGF. Moreover, the results of these macrophage depletion studies are consistent with the fact that M1 and M2 macrophages represent different and sometimes co-existing functional phenotypes of the same population. They polarize across their life span according to stimuli within the microenvironment in which they reside during the progression kidney diseases.

Opposing roles of phenotypically distinct macrophages suggested that targeting macrophages of different phenotypes may not be practical in developing therapeutic treatment for fibrotic diseases (Cao et al., 2014). More importantly, precise targeting of functionally different macrophages with opposing roles requires a better understanding of downstream signaling events and the diverse functions of multi-functional cytokines, such as TGF-β1 (Qiao et al., 2018), which although profibrotic contributes to suppression of inflammation and to tissue repair in kidney (Tang et al., 2019).

Instead of targeting specific functional phenotypes of macrophages, targeting a central factor in multiple profibrotic signaling pathways in macrophages is likely to be a more effective strategy for treating kidney diseases. Indeed, we found in the UUO model that inhibition of β-catenin/TCF promotes β-catenin/Foxo in the Wnt and TGF-β signaling pathways of bone marrow-derived macrophages (Yang et al., 2019). Importantly, redirection of β-catenin binding from TCF to Foxo resulted in reduction of inflammatory cytokines produced by bone marrow-derived macrophages, altered the fate of MMT macrophages and protected against kidney fibrosis (Yang et al., 2019).

## Conclusion

Macrophages are master regulators of inflammation and kidney fibrosis. Monocytes and macrophages are recruited and activated in response to chemoattractants and stimuli released after kidney injury. Macrophage plasticity adds complexity to their central roles in kidney fibrosis. After kidney injury, macrophages polarize into various phenotypes in response to alteration of the microenvironment in kidney disease. M1 pro-inflammatory macrophages clear infection but also cause kidney injury; M2 anti-inflammatory macrophages contribute to resolution of inflammation and kidney repair yet cause kidney fibrosis (Tang et al., 2019). Functionally distinct macrophage phenotypes contribute to the fibro-inflammatory microenvironment by abundant secretion of inflammatory and anti-inflammatory cytokines, mediators of tissue repair including TGF-β, Wnt ligands, PDGF, CTGF as well as all components of RAS. Those tissue repair mediators are also key inducers of kidney fibrosis when secreted in excess and maintained at higher levels in the chronic inflammatory milieu of kidney disease. Profibrotic mediators activate a profibrotic signaling network by cross-talking among multiple signaling pathways including TGF-β, Wnts, RAS, intergin/ILK, mTOR. Importantly, multiple pro-fibrotic signaling pathways all converge at activation of β-catenin/TCF, making β-catenin/TCF a key target for prevention of kidney fibrosis. Switching β-catenin/TCF to β-catenin/Foxo redirects signaling from profibrotic to anti-inflammatory and protects against kidney fibrosis. Targeting macrophages has long been proposed as a treatment for fibro-inflammatory kidney diseases. However, the phenotypic plasticity and conflicting roles of M2 macrophages are major hurdles for their therapeutic application. Recently we have identified the β-catenin/TCF/Foxo axis as a key determinant of the signaling direction of multiple profibrotic pathways. Thus, targeting macrophage signaling pathway via the β-catenin/TCF/Foxo axis may provide a new promising strategy for the treatment of kidney fibrosis in chronic kidney diseases.

## Author Contributions

JXu, JXie, DH, and GZ contributed to conception and design of the study. XW wrote the first draft of the manuscript. GZ, JC, JXu, DH, and JXie wrote sections of the manuscript. All authors contributed to manuscript revision, read, and approved the submitted version.

## Conflict of Interest

The authors declare that the research was conducted in the absence of any commercial or financial relationships that could be construed as a potential conflict of interest.

## Publisher’s Note

All claims expressed in this article are solely those of the authors and do not necessarily represent those of their affiliated organizations, or those of the publisher, the editors and the reviewers. Any product that may be evaluated in this article, or claim that may be made by its manufacturer, is not guaranteed or endorsed by the publisher.
